# Supplementary figures and images for: Analysis of changes in the occurrence of ice phenomena in upland and mountain rivers of Poland
Source: PLoS One. 2024 Jul 26;19(7):e0307842. doi: 10.1371/journal.pone.0307842 (PMC11280165; doi:10.1371/journal.pone.0307842)

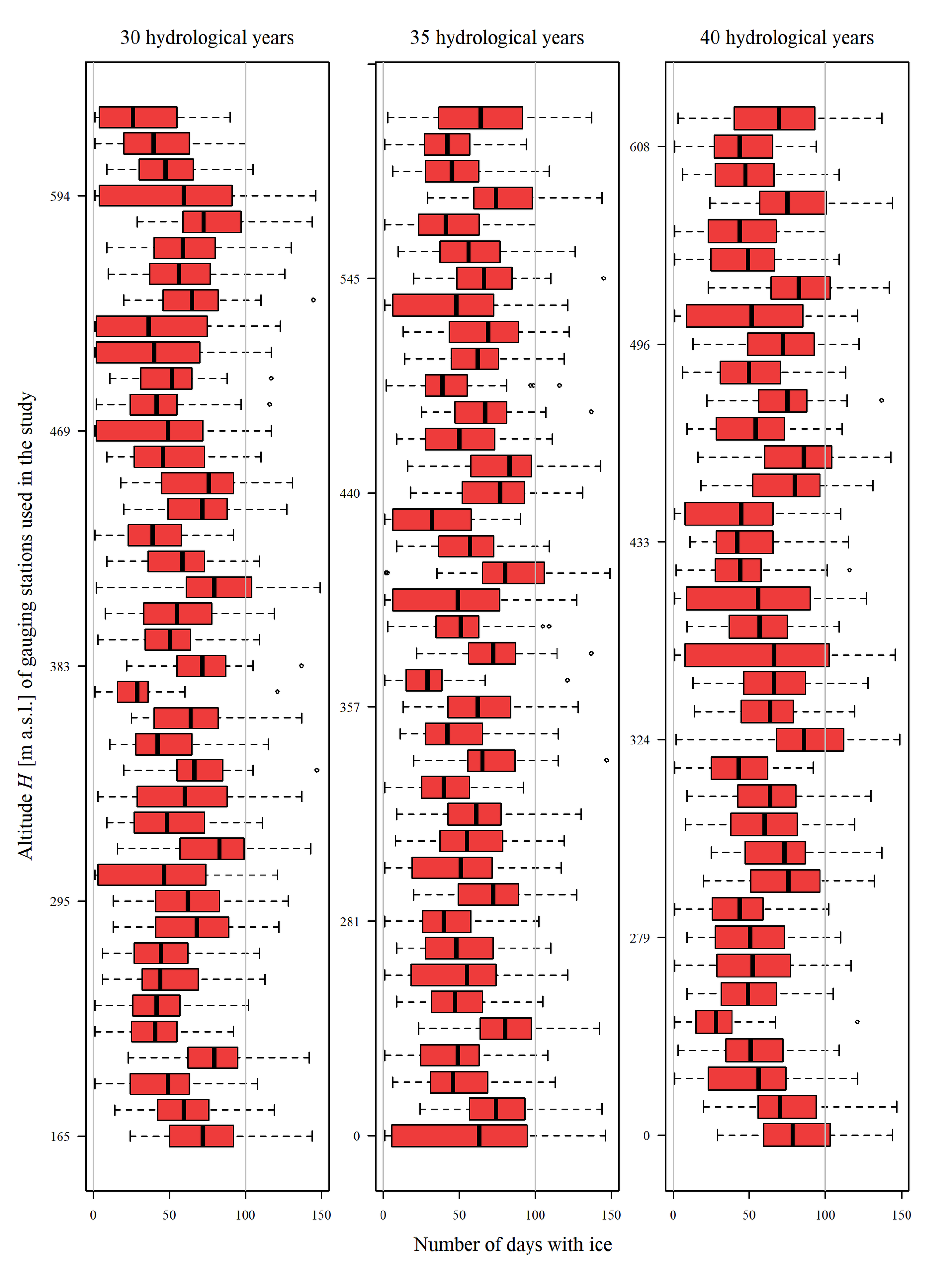

Supplement: S1 Fig — (TIF) [file pone.0307842.s001.tif]

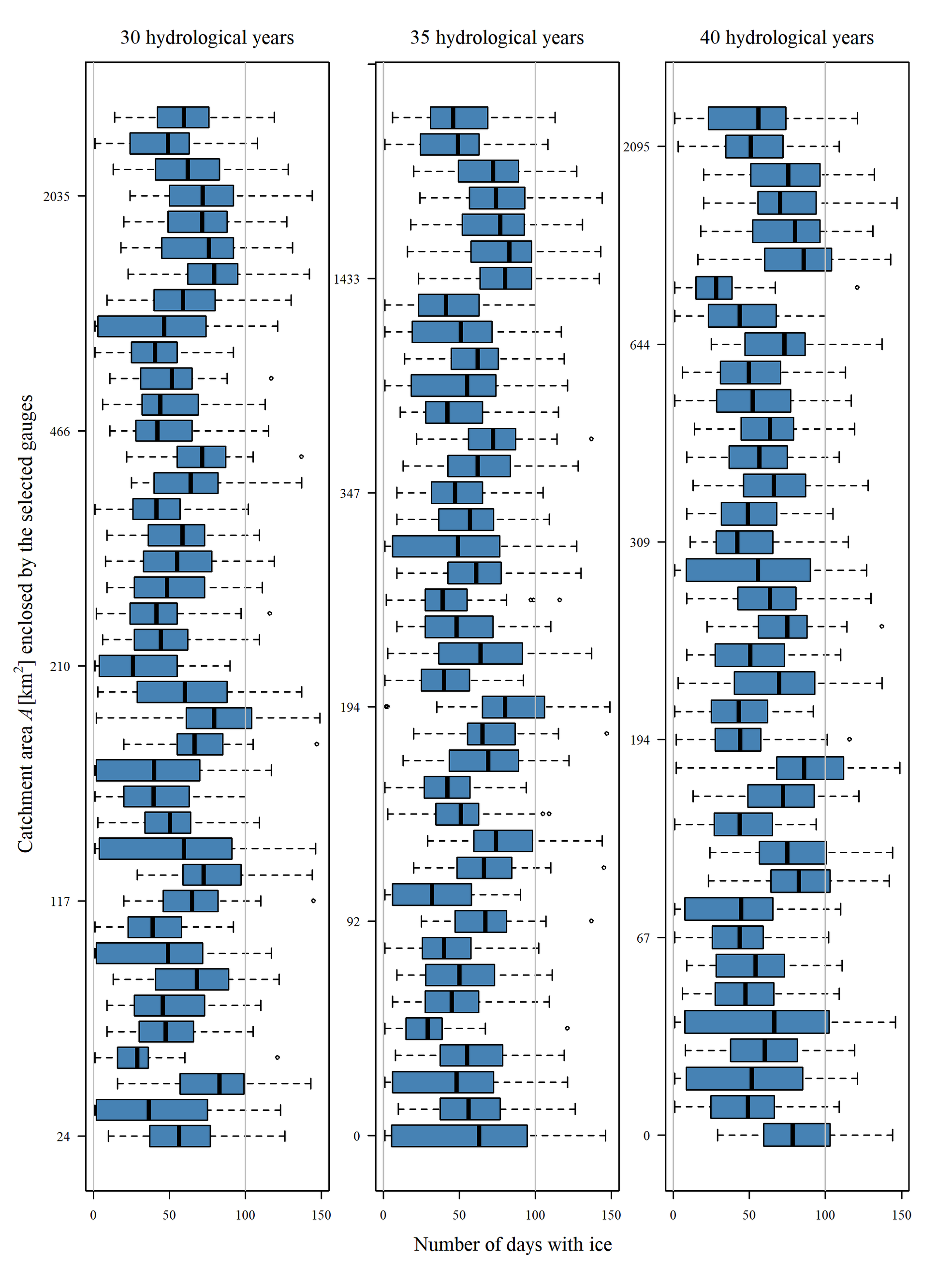

Supplement: S2 Fig — (TIF) [file pone.0307842.s002.tif]
