# Supplementary material for: Analysis of changes in the occurrence of ice phenomena in upland and mountain rivers of Poland
Source: PLoS One. 2024 Jul 26;19(7):e0307842. doi: 10.1371/journal.pone.0307842 (PMC11280165; doi:10.1371/journal.pone.0307842)
Supplement: S1 Appendix — (DOCX) [file pone.0307842.s004.docx]

Appendix A

**Table A1. Characteristics of gauging cross-sections and the catchments enclosed by those sections.**

| No | River/Gauging cross-section | Area,  *A*  [km^2^] | Altitude,  *H*  [m a.s.l] | Geographical coordinates | | Synoptic station | Average annual temperature (1992-2021), *t_a_* [ºC] | Average winter temperature (1992-2021), *t_aw_* [ºC] |
| --- | --- | --- | --- | --- | --- | --- | --- | --- |
|  |  |  |  | Longitude | Latitude |  |  |  |
| 1 | Biała/Ciężkowice | 524.6 | 238.5 | 20.973 | 49.792 | Nowy Sącz | 9.0 | 2.6 |
| 2 | Biała/Grybów | 207.0 | 320.5 | 20.946 | 49.624 | Tarnów | 9.4 | 3.0 |
| 3 | Biały Dunajec/Szaflary | 209.9 | 635.5 | 20.026 | 49.425 | Zakopane | 6.3 | 0.1 |
| 4 | Czarna/Polana | 94.1 | 437.4 | 22.574 | 49.302 | Lesko | 8.2 | 1.8 |
| 5 | Czarna Orawa/Jabłonka | 135.9 | 608.0 | 19.691 | 49.471 | Zakopane | 6.3 | 0.1 |
| 6 | Dunajec/Nowy Targ-Kowaniec | 686.9 | 573.5 | 20.054 | 49.487 | Zakopane | 6.3 | 0.1 |
| 7 | Jasiołka/Zboiska | 264.3 | 311.6 | 21.698 | 49.574 | Krosno | 8.6 | 2.1 |
| 8 | Kamienica/Łabowa | 64.9 | 450.2 | 20.859 | 49.527 | Nowy Sącz | 9.0 | 2.6 |
| 9 | Kamienica/Nowy Sącz | 237.0 | 278.8 | 20.696 | 49.625 | Nowy Sącz | 9.0 | 2.6 |
| 10 | Lepietnica/Ludźmierz | 50.3 | 596.9 | 19.976 | 49.469 | Zakopane | 6.3 | 0.1 |
| 11 | Łososina/Jakubkowice | 347.1 | 246.3 | 20.629 | 49.739 | Nowy Sącz | 9.0 | 2.6 |
| 12 | Łubinka/Nowy Sącz | 66.6 | 281.3 | 20.705 | 49.635 | Nowy Sącz | 9.0 | 2.6 |
| 13 | Niedziczanka/Niedzica | 136.7 | 495.7 | 20.302 | 49.411 | Zakopane | 6.3 | 0.1 |
| 14 | Olza/Istebna | 34.9 | 533.9 | 18.893 | 49.572 | Bielsko-Biała | 9.0 | 2.8 |
| 15 | Osława/Szczawne | 300.7 | 392.9 | 22.150 | 49.404 | Lesko | 8.2 | 1.8 |
| 16 | Pielnica/Nowosielce | 38.9 | 301.8 | 22.071 | 49.567 | Lesko | 8.2 | 1.8 |
| 17 | Poprad/Muszyna | 1518.8 | 446.3 | 20.892 | 49.339 | Nowy Sącz | 9.0 | 2.6 |
| 18 | Poprad/Muszyna-Milik | 1700.4 | 440.4 | 20.885 | 49.350 | Nowy Sącz | 9.0 | 2.6 |
| 19 | Poprad/Stary Sącz | 2075.0 | 295.3 | 20.660 | 49.568 | Nowy Sącz | 9.0 | 2.6 |
| 20 | Raba/Kasinka Mała | 353.3 | 356.9 | 20.033 | 49.705 | Zakopane | 6.3 | 0.1 |
| 21 | Raba/Rabka 2 | 91.9 | 469.0 | 19.948 | 49.613 | Kraków-Balice | 9.0 | 2.4 |
| 22 | Raba/Stróża | 644.2 | 297.0 | 19.925 | 49.796 | Zakopane | 6.3 | 0.1 |
| 23 | Ropa/Klęczany | 484.1 | 259.1 | 21.217 | 49.701 | Tarnów | 9.4 | 3.0 |
| 24 | San/Przemyśl | 3687.5 | 190.5 | 22.767 | 49.785 | Lesko | 8.2 | 1.8 |
| 25 | San/Zatwarnica | 494.2 | 486.2 | 22.563 | 49.235 | Lesko | 8.2 | 1.8 |
| 26 | Skawa/Sucha Beskidzka | 466.1 | 324.0 | 19.609 | 49.739 | Kraków-Balice | 9.0 | 2.4 |
| 27 | Solinka/Terka | 309.1 | 432.8 | 22.429 | 49.300 | Lesko | 8.2 | 1.8 |
| 28 | Soła/Cięcina | 413.6 | 383.1 | 19.149 | 49.625 | Bielsko-Biała | 9.0 | 2.8 |
| 29 | Soła/Rajcza | 253.8 | 482.0 | 19.116 | 49.514 | Bielsko-Biała | 9.0 | 2.8 |
| 30 | Strwiąż/Krościenko | 194.4 | 400.7 | 22.663 | 49.473 | Lesko | 8.2 | 1.8 |
| 31 | Stryszawka/Sucha Beskidzka | 140.5 | 323.9 | 19.601 | 49.743 | Kraków-Balice | 9.0 | 2.4 |
| 32 | Tanew/Harasiuki | 2034.8 | 164.5 | 22.474 | 50.478 | Rzeszów-Jasionka | 8.9 | 2.3 |
| 33 | Wetlina/Kalnica | 119.0 | 573.7 | 22.429 | 49.189 | Lesko | 8.2 | 1.8 |
| 34 | Wielki Rogoźnik/Ludźmierz | 124.3 | 594.0 | 19.987 | 49.459 | Zakopane | 6.3 | 0.1 |
| 35 | Wilczka/Wilkanów | 46.7 | 362.8 | 16.662 | 50.265 | Kłodzko | 8.2 | 2.1 |
| 36 | Wisłok/Puławy | 130.0 | 384.9 | 21.907 | 49.491 | Krosno | 8.6 | 2.1 |
| 37 | Wisłok/Żarnowa | 1433.0 | 213.5 | 21.818 | 49.878 | Krosno | 8.6 | 2.1 |
| 38 | Wisłoka/Krajowice | 2095.4 | 213.4 | 21.413 | 49.771 | Krosno | 8.6 | 2.1 |
| 39 | Wołosaty/Stuposiany | 116.9 | 544.5 | 22.684 | 49.186 | Lesko | 8.2 | 1.8 |
| 40 | Żabniczanka/Żabnica | 23.9 | 564.8 | 19.180 | 49.564 | Bielsko-Biała | 9.0 | 2.8 |

**Table A2. The results of the trend analysis for average annual temperature *t_a_* [ºC] and average winter annual temperature *t_aw_* [ºC]; *p_v_* means *p*-value of the Mann-Kendall test, asterisk ↓ shows a downward trend, and ▪ - no trend.**

| No | River/Gauging cross-section | 30 hydrological years, 1992-2021 | | | | 35 hydrological years, 1987-2021 | | | | 40 hydrological years, 1982-2021 | | | | 50 hydrological years, 1972-2021 | | | |
| --- | --- | --- | --- | --- | --- | --- | --- | --- | --- | --- | --- | --- | --- | --- | --- | --- | --- |
|  |  | *t_a_* [ºC] | | *t_aw_* [ºC] | | *t_a_* [ºC] | | *t_aw_* [ºC] | | *t_a_* [ºC] | | *t_aw_* [ºC] | | *t_a_* [ºC] | | *t_aw_* [ºC] | |
|  |  | *p_v_* | trend | *p_v_* | trend | *p_v_* | trend | *p_v_* | trend | *p_v_* | trend | *p_v_* | trend | *p_v_* | trend | *p_v_* | trend |
| 1 | Biała/Ciężkowice | 0.003 | ↑ | 0.033 | ↑ |  |  |  |  |  |  |  |  |  |  |  |  |
| 2 | Biała/Grybów | 0.005 | ↑ | 0.033 | ↑ | 0.003 | ↑ | 0.033 | ▪ | 0.000 | ↑ | 0.016 | ↑ |  |  |  |  |
| 3 | Biały Dunajec/Szaflary | 0.004 | ↑ | 0.033 | ↑ | 0.005 | ↑ | 0.033 | ▪ | 0.000 | ↑ | 0.010 | ↑ |  |  |  |  |
| 4 | Czarna/Polana | 0.003 | ↑ | 0.033 | ↑ | 0.004 | ↑ | 0.033 | ▪ | 0.000 | ↑ | 0.010 | ↑ |  |  |  |  |
| 5 | Czarna Orawa/Jabłonka | 0.004 | ↑ | 0.033 | ↑ | 0.003 | ↑ | 0.033 | ▪ | 0.000 | ↑ | 0.010 | ↑ |  |  |  |  |
| 6 | Dunajec/Nowy Targ-Kowaniec | 0.004 | ↑ | 0.033 | ↑ | 0.004 | ↑ | 0.033 | ▪ | 0.000 | ↑ | 0.010 | ↑ |  |  |  |  |
| 7 | Jasiołka/Zboiska | 0.003 | ↑ | 0.033 | ↑ | 0.004 | ↑ | 0.033 | ▪ | 0.000 | ↑ | 0.010 | ↑ |  |  |  |  |
| 8 | Kamienica/Łabowa | 0.005 | ↑ | 0.033 | ↑ | 0.003 | ↑ | 0.033 | ▪ | 0.000 | ↑ | 0.016 | ↑ |  |  |  |  |
| 9 | Kamienica/Nowy Sącz | 0.005 | ↑ | 0.033 | ↑ | 0.005 | ↑ | 0.033 | ▪ | 0.000 | ↑ | 0.016 | ↑ |  |  |  |  |
| 10 | Lepietnica/Ludźmierz | 0.004 | ↑ | 0.033 | ↑ | 0.005 | ↑ | 0.033 | ▪ | 0.000 | ↑ | 0.010 | ↑ |  |  |  |  |
| 11 | Łososina/Jakubkowice | 0.005 | ↑ | 0.033 | ↑ | 0.004 | ↑ | 0.033 | ▪ | 0.000 | ↑ | 0.016 | ↑ |  |  |  |  |
| 12 | Łubinka/Nowy Sącz | 0.005 | ↑ | 0.033 | ↑ | 0.005 | ↑ | 0.033 | ▪ | 0.000 | ↑ | 0.016 | ↑ |  |  |  |  |
| 13 | Niedziczanka/Niedzica | 0.004 | ↑ | 0.033 | ↑ | 0.005 | ↑ | 0.033 | ▪ | 0.000 | ↑ | 0.010 | ↑ |  |  |  |  |
| 14 | Olza/Istebna | 0.004 | ↑ | 0.033 | ↑ | 0.004 | ↑ | 0.033 | ▪ | 0.000 | ↑ | 0.010 | ↑ |  |  |  |  |
| 15 | Osława/Szczawne | 0.003 | ↑ | 0.033 | ↑ | 0.004 | ↑ | 0.033 | ▪ | 0.000 | ↑ | 0.010 | ↑ |  |  |  |  |
| 16 | Pielnica/Nowosielce | 0.003 | ↑ | 0.033 | ↑ | 0.003 | ↑ | 0.033 | ▪ | 0.000 | ↑ | 0.010 | ↑ |  |  |  |  |
| 17 | Poprad/Muszyna | 0.005 | ↑ | 0.033 | ↑ | 0.003 | ↑ | 0.033 | ▪ | 0.000 | ↑ | 0.016 | ↑ |  |  |  |  |
| 18 | Poprad/Muszyna-Milik | 0.005 | ↑ | 0.033 | ↑ | 0.005 | ↑ | 0.033 | ▪ | 0.000 | ↑ | 0.016 | ↑ |  |  |  |  |
| 19 | Poprad/Stary Sącz | 0.005 | ↑ | 0.033 | ↑ | 0.005 | ↑ | 0.033 | ▪ | 0.000 | ↑ | 0.016 | ↑ |  |  |  |  |
| 20 | Raba/Kasinka Mała | 0.004 | ↑ | 0.033 | ↑ | 0.005 | ↑ | 0.033 | ▪ | 0.000 | ↑ | 0.010 | ↑ |  |  |  |  |
| 21 | Raba/Rabka 2 | 0.004 | ↑ | 0.033 | ↑ | 0.004 | ↑ | 0.033 | ▪ |  |  |  |  |  |  |  |  |
| 22 | Raba/Stróża | 0.011 | ↑ | 0.058 | ▪ | 0.004 | ↑ | 0.033 | ▪ | 0.001 | ↑ | 0.021 | ↑ |  |  |  |  |
| 23 | Ropa/Klęczany | 0.003 | ↑ | 0.033 | ↑ | 0.011 | ↑ | 0.058 | ▪ | 0.000 | ↑ | 0.016 | ↑ | 0.000 | ↑ | 0.010 | ↑ |
| 24 | San/Przemyśl | 0.003 | ↑ | 0.033 | ↑ | 0.003 | ↑ | 0.033 | ▪ | 0.000 | ↑ | 0.010 | ↑ |  |  |  |  |
| 25 | San/Zatwarnica | 0.003 | ↑ | 0.033 | ↑ | 0.003 | ↑ | 0.033 | ▪ | 0.000 | ↑ | 0.010 | ↑ |  |  |  |  |
| 26 | Skawa/Sucha Beskidzka | 0.011 | ↑ | 0.058 | ▪ | 0.003 | ↑ | 0.033 | ▪ | 0.001 | ↑ | 0.021 | ↑ |  |  |  |  |
| 27 | Solinka/Terka | 0.003 | ↑ | 0.033 | ↑ | 0.011 | ↑ | 0.058 | ▪ | 0.000 | ↑ | 0.010 | ↑ |  |  |  |  |
| 28 | Soła/Cięcina | 0.004 | ↑ | 0.033 | ↑ | 0.003 | ↑ | 0.033 | ▪ | 0.000 | ↑ | 0.010 | ↑ |  |  |  |  |
| 29 | Soła/Rajcza | 0.004 | ↑ | 0.033 | ↑ | 0.004 | ↑ | 0.033 | ▪ | 0.000 | ↑ | 0.010 | ↑ |  |  |  |  |
| 30 | Strwiąż/Krościenko | 0.003 | ↑ | 0.033 | ↑ | 0.004 | ↑ | 0.033 | ▪ | 0.000 | ↑ | 0.010 | ↑ |  |  |  |  |
| 31 | Stryszawka/Sucha Beskidzka | 0.011 | ↑ | 0.058 | ▪ | 0.003 | ↑ | 0.033 | ▪ | 0.001 | ↑ | 0.021 | ↑ |  |  |  |  |
| 32 | Tanew/Harasiuki | 0.001 | ↑ | 0.033 | ↑ | 0.011 | ↑ | 0.058 | ▪ | 0.000 | ↑ | 0.010 | ↑ |  |  |  |  |
| 33 | Wetlina/Kalnica | 0.003 | ↑ | 0.033 | ↑ | 0.001 | ↑ | 0.033 | ▪ | 0.000 | ↑ | 0.010 | ↑ |  |  |  |  |
| 34 | Wielki Rogoźnik/Ludźmierz | 0.004 | ↑ | 0.033 | ↑ | 0.003 | ↑ | 0.033 | ▪ | 0.000 | ↑ | 0.010 | ↑ |  |  |  |  |
| 35 | Wilczka/Wilkanów | 0.011 | ↑ | 0.033 | ↑ | 0.004 | ↑ | 0.033 | ▪ | 0.001 | ↑ | 0.010 | ↑ |  |  |  |  |
| 36 | Wisłok/Puławy | 0.003 | ↑ | 0.033 | ↑ | 0.011 | ↑ | 0.033 | ▪ |  |  |  |  |  |  |  |  |
| 37 | Wisłok/Żarnowa | 0.003 | ↑ | 0.033 | ↑ | 0.003 | ↑ | 0.033 | ▪ | 0.000 | ↑ | 0.010 | ↑ |  |  |  |  |
| 38 | Wisłoka/Krajowice | 0.003 | ↑ | 0.033 | ↑ | 0.003 | ↑ | 0.033 | ▪ | 0.000 | ↑ | 0.016 | ↑ |  |  |  |  |
| 39 | Wołosaty/Stuposiany | 0.003 | ↑ | 0.033 | ↑ | 0.003 | ↑ | 0.033 | ▪ | 0.000 | ↑ | 0.010 | ↑ |  |  |  |  |
| 40 | Żabniczanka/Żabnica | 0.004 | ↑ | 0.033 | ↑ | 0.003 | ↑ | 0.033 | ▪ | 0.000 | ↑ | 0.010 | ↑ |  |  |  |  |
