# Supplementary material for: Analysis of changes in the occurrence of ice phenomena in upland and mountain rivers of Poland
Source: PLoS One. 2024 Jul 26;19(7):e0307842. doi: 10.1371/journal.pone.0307842 (PMC11280165; doi:10.1371/journal.pone.0307842)
Supplement: Attachment — Submitted filename: Answer to Reviewers.docx [file pone.0307842.s005.docx]

**Answers to the Reviewers’ remarks**

Dear Editors, dear Reviewers,

We would like to thank very much for the remarks and your valuable time you devoted to review our paper. We believe, that thanks to your remarks the new version, which was significantly reconstructed, will meet the high standards of the PLOS ONE. Since there were ten reviewers we decided to address the remarks in the table, which is, in our opinion, the most convenient way of organisation of the remarks and answers.

We count that the new version of the manuscript will satisfy both the Reviewers, Editors and finally the readers.

With best regards

Yours faithfully

Authors

Edirors’ remarks

| Remark | Answer |
| --- | --- |
| Please ensure that your manuscript meets PLOS ONE's style requirements, including those for file naming. The PLOS ONE style templates can be found at [https://journals.plos.org/plosone/s/file?id=wjVg/PLOSOne_formatting_sample_main_body.pdf](https://journals.plos.org/plosone/s/file?id=wjVg/PLOSOne_formatting_sample_main_body.pdf) and [https://journals.plos.org/plosone/s/file?id=ba62/PLOSOne_formatting_sample_title_authors_affiliations.pdf](https://journals.plos.org/plosone/s/file?id=ba62/PLOSOne_formatting_sample_title_authors_affiliations.pdf) | We thank the editors for this remark – the paper has been doublechecked according to the PLOS ONE style templates. |
| Please upload a copy of Figures 8-14, to which you refer in your text on your PDF file. If the figure is no longer to be included as part of the submission please remove all reference to it within the text. | We do not quite understand the Editor’s remark. We have only 9 figures: Figs 1-7 and 2 in the appendix. |
| We note that [Figure 1,2,3,4 and 6a-c] in your submission contain [map/satellite] images which may be copyrighted. All PLOS content is published under the Creative Commons Attribution License (CC BY 4.0), which means that the manuscript, images, and Supporting Information files will be freely available online, and any third party is permitted to access, download, copy, distribute, and use these materials in any way, even commercially, with proper attribution. For these reasons, we cannot publish previously copyrighted maps or satellite images created using proprietary data, such as Google software (Google Maps, Street View, and Earth). For more information, see our copyright guidelines: [http://journals.plos.org/plosone/s/licenses-and-copyright](http://journals.plos.org/plosone/s/licenses-and-copyright).  We require you to either (1) present written permission from the copyright holder to publish these figures specifically under the CC BY 4.0 license, or (2) remove the figures from your submission:  a. You may seek permission from the original copyright holder of Figure 1,2,3,4 and 6a-c to publish the content specifically under the CC BY 4.0 license.  We recommend that you contact the original copyright holder with the Content Permission Form ([http://journals.plos.org/plosone/s/file?id=7c09/content-permission-form.pdf](http://journals.plos.org/plosone/s/file?id=7c09/content-permission-form.pdf)) and the following text: “I request permission for the open-access journal PLOS ONE to publish XXX under the Creative Commons Attribution License (CCAL) CC BY 4.0 ([http://creativecommons.org/licenses/by/4.0/](http://creativecommons.org/licenses/by/4.0/)). Please be aware that this license allows unrestricted use and distribution, even commercially, by third parties. Please reply and provide explicit written permission to publish XXX under a CC BY license and complete the attached form.”  Please upload the completed Content Permission Form or other proof of granted permissions as an "Other" file with your submission.  In the figure caption of the copyrighted figure, please include the following text: “Reprinted from [ref] under a CC BY license, with permission from [name of publisher], original copyright [original copyright year].”  b. If you are unable to obtain permission from the original copyright holder to publish these figures under the CC BY 4.0 license or if the copyright holder’s requirements are incompatible with the CC BY 4.0 license, please either i) remove the figure or ii) supply a replacement figure that complies with the CC BY 4.0 license. Please check copyright information on all replacement figures and update the figure caption with source information. If applicable, please specify in the figure caption text when a figure is similar but not identical to the original image and is therefore for illustrative purposes only. The following resources for replacing copyrighted map figures may be helpful:  USGS National Map Viewer (public domain): [http://viewer.nationalmap.gov/viewer/](http://viewer.nationalmap.gov/viewer/) The Gateway to Astronaut Photography of Earth (public domain): [http://eol.jsc.nasa.gov/sseop/clickmap/](http://eol.jsc.nasa.gov/sseop/clickmap/) Maps at the CIA (public domain): [https://www.cia.gov/library/publications/the-world-factbook/index.html](https://www.cia.gov/library/publications/the-world-factbook/index.html) and [https://www.cia.gov/library/publications/cia-maps-publications/index.html](https://www.cia.gov/library/publications/cia-maps-publications/index.html) NASA Earth Observatory (public domain): [http://earthobservatory.nasa.gov/](http://earthobservatory.nasa.gov/) Landsat: [http://landsat.visibleearth.nasa.gov/](http://landsat.visibleearth.nasa.gov/) USGS EROS (Earth Resources Observatory and Science (EROS) Center) (public domain): [http://eros.usgs.gov/#](http://eros.usgs.gov/) Natural Earth (public domain): [http://www.naturalearthdata.com/](http://www.naturalearthdata.com/) | All maps were created by the authors in the free QGIS 3.20.3 software. We have not used Google software (Google Maps, Street View, and Earth).  Indeed, to draw Figure 2, we used the background from the geoportal system.  The geoportal is free and publicly available, and using it only requires quoting the geoportal.  So we quoted “The hypsometric map (DEM) is available at: [https://www.geoportal.gov.pl/pl/usluga/uslugi-przegladania-wms-i-wmts](https://www.geoportal.gov.pl/pl/usluga/uslugi-przegladania-wms-i-wmts) (accessed on 3 April 2024)”. |

Reviewer #1

| Remark | Answer |
| --- | --- |
| In summary, the research titled 'Trends in Ice Phenomena on Polish Rivers' sheds light on the current state of ice formation on rivers in Poland. The findings reveal a concerning increase in the frequency and severity of ice phenomena, which have negative consequences for the environment, transportation, and economic activities. Given the originality, scientific rigor, and contribution to existing knowledge, it is recommended that the report be published in a peer-reviewed scientific. This will allow for a wider dissemination of the research findings and foster meaningful discussions on the topic of ice phenomena on rivers. The study's use of both quantitative and qualitative methods adds strength to the results, and it adds to the existing understanding of the impact of climate change on water resources in Central and Eastern Europe. Furthermore, the report suggests further research to explore the underlying causes of the observed trends and their specific effects on different sectors and regions in Poland. Ultimately, publishing this research in a reputable scientific will enhance its reach and significance in ongoing discussions on this subject. | We would like to thank very much the Reviewer for his/her kind words about our work. We, nevertheless, introduced some amendments to improve the clarity of the paper (see below). |
| The research has yielded important insights that make it essential for the report to be published in a peer-reviewed scientific. This recommendation is supported by the following reasons:  Firstly, the research is both original and relevant. It offers valuable information on the current trends of ice formation on Polish rivers, a topic that has not been extensively studied. The findings are of great importance to policymakers, environmentalists, and other stakeholders involved in managing the impacts of ice phenomena on rivers.  Secondly, the research was conducted with a high level of scientific rigor.  A combination of qualitative and quantitative methods were used to collect and analyze data, enhancing the reliability and validity of the findings. The use of statistical tools such as trend analysis and regression analysis further strengthens the credibility of the research. Moreover, the research contributes to the existing body of knowledge on ice phenomena on rivers, particularly in Poland. It provides new evidence that can either support or challenge existing theories and hypotheses on the subject. This further highlights the importance of publishing the report in a peer-reviewed scientific.  Lastly, the research findings have significant policy implications for the management of ice phenomena on Polish rivers. The results can be utilized to inform the development of effective policies and strategies for mitigating the potential risks associated with ice formation. Therefore, publishing the report in a peer-reviewed scientific is crucial to ensure that the findings reach the appropriate audience and have a positive impact on policy-making. |  |

Reviewer #2

| Remark | Answer |
| --- | --- |
| Country name should be added to the name of the article. | We thank the reviewer for the kind remark. We changed the title accordingly. |
| The written language of the article must be in academic language. (For example, the word "we" should not be used). | The whole text has been corrected and sentences containing personal articles have been rephrased. |
| Numerical results of the analysis results should be given in the abstract section of the article. | Several numerical results have been added to the abstract. In general, the title of the article and the abstract have been changed significantly, both in response to the reviewers' comments as well as in order to adapt to the extended scope of studies in the new version of the article. An analysis of meteorological factors, such as annual and winter (Nov-Apr) air temperature, precipitation, and water temperature, was carried out. Moreover, the relationships between the occurrence of ice phenomena and the above-mentioned factors were examined. We believe that the revised title and abstract better describe the current study. Generic statements and those weakly related to actual research have been removed. |
| Studies on both climate change and trends in different countries can be added to the literature section of the article. A few examples of study are given below. - [https://www.sciencedirect.com/science/article/abs/pii/S0960148124001423?via%3Dihub](https://www.sciencedirect.com/science/article/abs/pii/S0960148124001423?via%3Dihub) - [https://link.springer.com/article/10.1007/s10661-023-11236-3](https://link.springer.com/article/10.1007/s10661-023-11236-3) - [https://link.springer.com/article/10.1007/s00477-021-02067-0](https://link.springer.com/article/10.1007/s00477-021-02067-0) - [https://iwaponline.com/jwcc/article/13/6/2278/88161/GCMs-simulation-based-assessment-for-the-response](https://iwaponline.com/jwcc/article/13/6/2278/88161/GCMs-simulation-based-assessment-for-the-response) - [https://journals.ametsoc.org/view/journals/apme/61/12/JAMC-D-22-0081.1.xml](https://journals.ametsoc.org/view/journals/apme/61/12/JAMC-D-22-0081.1.xml) | The ‘Introduction’ section has been significantly revised in line with reviewers' comments. We shortened the introduction by removing unnecessary fragments poorly related to the scope of research and arranged the issues so that we first presented a global overview and then a local perspective. We have corrected and clarified the results of the research described in the literature review, we have added appropriate numerical values confirming the presented conclusions from the discussed articles. We paid particular attention to a precise review of the literature regarding Poland. We also added information about new methodological approaches used lately for detecting trends in hydro-meteorological variables. Finally, we have changed the goal of the paper to be consistent with the reviewers' comments and with the expanded scope of research in the new version of the article.  One of the proposed papers has been cited in relation to new methodological approaches used lately for detecting trends. The others consider rather the warm climatic zones, where the problem of ice disappearance is not considered. |
| The image quality of all graphics in the article is very low.  The quality of the figures was improved (tiff file, min 300 dpi). | Indeed, the image quality in the PDF produced by the PLOS ONE’s submission system is poor, but we have no influence on it. Therefore, the system provides the possibility to download the figures in their full resolution (we previously uploaded figures in 600 dpi tiff format) – see the link in the top corner of the page by each figure. Nevertheless, the figures were corrected, redesigned and uploaded in 300 dpi resolution. |
| Can a homogeneity test be applied to data? | Yes, when we consider a time series to be homogenous if all the random variables the series consists of have the same location parameters, then the Mann-Kendall (MK) test is a commonly used method of testing homogeneity. |
| Can one of the modern trend methods (such as ITA, ITTA, IPTA, 3D-ITA, ITPAM) be applied to the article's data? It may be useful to compare analysis results. | We fully agree with the Reviewer’s suggestion that novel methods can be applied to the data. But, please note that the aim of this article was to analyse datasets by the commonly used and accepted methods, not the comparison of approaches and the results obtained by different methods. To compare all the methods you listed + the MK test in application to the ‘number of days with ice’ data is an extensive study that lies beyond the main scope of the paper. The properties of the methods should be evaluated before application, e.g. the size and power of the tests when applied to the ’number of days with ice’ data. Such an assessment is necessary to be sure that conclusions of hypothesis testing are justified. We did an extensive literature review but we did not find such a study. Therefore, we decided to use only the MK test the good size and power properties of which are well known, and the methods you mentioned can be evaluated in the future. |
| Why is the table empty for 50 hydrological years? | The Table has been corrected, i.e. the almost empty columns have been removed, and information about the trend for the 50-element data series has been added in the text. |
| In the conclusion section of the article, suggestions, weaknesses and strengths of this study, and what the next study should be like should be included. | We thank the Reviewer for this kind suggestion. A short paragraph addressing this suggestion has been added to the text. |

Reviewer #3

| Remark | Answer |
| --- | --- |
| Though the results presented in the manuscript (ice-day trend results) are interesting, the manuscript requires major revisions. In particular, climate (temperature and precipitation) time series should also be analyzed. | We agree that identifying the causes of the decrease in the number of days with ice makes the study more interesting. Therefore we downloaded additional climate data records from the IMGW-PIB repository (IMGW-PIB is the acronym of the Official Polish Hydrological Service). Details are given below. |
| The figure quality and literature review should also be improved. Please see below my detailed comments. | Indeed, the image quality in the PDF produced by the PLOS ONE’s submission system is poor, but we have no influence on it. Therefore, the system provides the possibility to download the figures in their full resolution (we previously uploaded figures in 600 dpi tiff format) – see the link in the top corner of the page by each figure. Nevertheless, the figures were corrected, redesigned and uploaded in 300 dpi resolution.  We corrected the literature review part in the manuscript. |
| The title should be more comprehensive. Something like “Analyzing Trends in Climate and Ice-Day Time Series along Polish Rivers”  Then you can show the analysis of at least temperature time series. For example, you may use MODIS land surface temperature at 1km and 6-day resolutions from [https://lpdaac.usgs.gov/products/mod11a2v061/](https://lpdaac.usgs.gov/products/mod11a2v061/) OR use the local climate data, etc. | The title of the article and the abstract have been changed significantly, both in response to the reviewers' comments as well as in order to adapt to the extended scope of studies in the new version of the article. An analysis of meteorological factors, such as annual and winter (Nov-Apr) air temperature, precipitation, and water temperature, was carried out. Moreover, the relationships between the occurrence of ice phenomena and the above-mentioned factors were examined. We believe that the revised title and abstract better describe the current study. Numerical results have been added to the abstract. Generic statements and those weakly related to actual research have been removed.  Air temperature and precipitation data are available from two kinds of stations through the IMGW-PIB repository: the synoptic (SY) and the climatological (CL) stations. The network of the SY and CL stations is relatively dense in the study area thus we did not use any other data source like MODIS. As stated in the Section 2.1 of the paper, there were 40 ‘ice stations’ (IS – a station where ice phenomena was observed) with data from the period 1992-2021, 39 ISs with data from 1987-2021, etc. Therefore, we used the SY and CL data for every time span, namely 1992-2021 (30 years), 1987-2021 (35 years), 1982-2021 (40 years), 1972-2021 (40 years), 1960-2021 (62 years), 1951-2017 (67 years).  For each time span, the procedure was as follows:   (1) For every IS two stations were identified: the nearest SY station and the nearest CL station (SY1 and CL1, Euclidean distance). Some ISs have the same SY1 or CL1 station. 9 synoptic and 11 climatological stations were identified at this step. The average distance from the IS station to the SY1 / CL1 station was 26.2 / 30.5 km; (2) Daily air temperature and daily precipitation data were downloaded for all SY1 and CL1 stations selected in (1); (3) Based on the data from the SY1 and CL1 stations, the series of climate characteristics of air temperature and precipitation were derived: the mean annual temp., the mean winter (Nov-Apr) temp., the minimum winter temp., the annual number of days with negative temp., the annual precipitation total, the annual maximum daily precip. total; (4) For each series of climate characteristics derived in (3), the one-sided hypothesis about trend was formulated and verified using the MK test.   The procedure (1-4) was repeated for every time span.  In results, the following trends were detected: the increasing in the mean annual temp. (all stations), increasing in the mean winter temp. (almost all stations), decreasing in the number of days with negative temp (50% of the stations), and increasing in the min winter temp. (several stations). No trend was observed in precipitation series.  In the paper, we described the results only for SY1 stations as they were similar at the SY1 and CL1 stations.  We also analysed water temperature. However, such data are not common in the IMGW-PIB databases. We identified only several stations (among the all studied stations) with water temperature data. Only three of them have full data records in 1992:2021. The mean annual winter temperature and the mean winter temperature were analysed. The increasing trend was detected in both variables (one station), in the mean annual temp. (two stations), and in the mean winter temp. (one stations).  The results were placed in the Results section. |
| The literature review should be improved. More recent works on trend analyses of water flow in cold climate regions should be added. For example, in Line 85, you may add Zaghloul et al. (2022) utilized water flow time series across rivers in cold climate region of Northern Canada using Mann-Kendall test and Sen’s slope, and they showed that winter water flow in the mountainous region has been rising gradually since 1956 due to temperature increase and gradual melting of snowpacks and glaciers. DOI: 10.3390/hydrology9110197  Also, the following review article by Wang et al (2022) on modelling watershed and river basin processes on cold climate region can be added: Doi: 10.3390/w13040518 | The ‘Introduction’ section has been significantly revised in line with reviewers' comments. We shortened the introduction by removing unnecessary fragments poorly related to the scope of research and arranged the issues so that we first presented a global overview and then a local perspective. We have corrected and clarified the results of the research described in the literature review, we have added appropriate numerical values confirming the presented conclusions from the discussed articles. We paid particular attention to a precise review of the literature regarding Poland. We also added information about new methodological approaches used lately for detecting trends in hydro-meteorological variables. Finally, we have changed the goal of the paper to be consistent with the reviewers' comments and with the expanded scope of research in the new version of the article.  We also mentioned about studies on the variability of hydro-meteorological factors in cold regions and one of the above papers has been cited in the paper. |
| Table 2. At what level are these slopes statistically significant? Is it at 99% or 95%? Please see the first article above that I mentioned above for more details. Please also discuss your results in line with their results in the discussion section. | The p-value applied that we considered appropriate to state the statistical significance of trends was 0.05. It was cearly stated in the text. |
| Figures are not professionally produced. Their quality and resolution should be improved. | Indeed, the image quality in the PDF produced by the PLOS ONE’s submission system is poor, but we have no influence on it. Therefore, the system provides the possibility to download the figures in their full resolution (we previously uploaded figures in 600 dpi tiff format) – see the link in the top corner of the page by each figure. Nevertheless, the figures were corrected, redesigned and uploaded in 300 dpi resolution. |
| Figure 2. The background color for elevation is not showing different elevation ranges, i.e., almost everywhere is greenish. I suggest adjusting the value range of color bar, so elevation can be better separated and visualized. Please see the first article that I suggested above as a guide on how to improve your figures. | The Figure 2 was corrected accordingly. Note that Poland is a lowland country (green), except the small mountaineering part in the south (yellowish and reddish). Unfortunately we cannot change the colour scheme of the map, because this is the only map which we downloaded as a ready product from the geoportal: [https://www.geoportal.gov.pl/pl/usluga/uslugi-przegladania-wms-i-wmts](https://www.geoportal.gov.pl/pl/usluga/uslugi-przegladania-wms-i-wmts) (accessed on 3 April 2024)”. |
| Lines 380-385. Including the analysis of precipitation and temperature is strongly recommended. For example, warming trend (if you found) can be linked to reduction of ice days. There are several methods of investigating the relationship between climate and ice days, e.g., least-squares triple cross-wavelet analysis and multivariate regression analysis. I suggest authors have look at these techniques and discuss. | Thank you for suggesting this. We decided to perform the regression analysis. However, because only temperature turned out to be the main cause of the decrease in the number of days with ice while precipitation was not, we used the following model: $x=at+b+\varepsilon$, where $x$ - the number of days with ice, $t$ - the mean winter temperature at the nearest synoptic station, and $\varepsilon$ – residuals. The results were placed in the section 3.2. Correlation and regression analysis. |

Reviewer #4

| Remark | Answer |
| --- | --- |
| The primary factor determining the form and duration of ice phenomena on rivers is the air temperature, the variability of which depends on atmospheric circulation. Air temperature fluctuations determine the variability in thermal conditions for waters in a given catchment area, and consequently have a direct impact on the formation of various ice forms on rivers and water reservoirs. Still, the paper lacked detailed elaboration on the geographical location, climate characteristics, and hydrological conditions of the study area, and did not analyze and compare the autocorrelation or trends of individual parameters such as air and water temperatures and ice phenomena during different series of years, so the conclusion that “The analysis of data of ice phenomena in southern Poland showed a large impact of climate change on ice in the temperate climate zone” (in line 432) is not convincing. | The climate characteristics such as mean annual temp. and mean winter temp. from the nearest synoptic stations (years 1992:2021) were added in Table A1.  Moreover, the trend analysis was carried out for (a) air temperature and precipitation, and (b) water temperature.   (a) air temperature and precipitation data are available from two kinds of stations through the IMGW-PIB repository: the synoptic (SY) and the climatological (CL) stations. The network of the SY and CL stations is relatively dense in the study area. As stated in the Section 2.1 of the paper, there were 40 ‘ice stations’ (IS – station where ice was observed) with data from the period 1992-2021, 39 ISs with data from 1987-2021, etc. Therefore, we used the SY and CL data for every time span, namely 1992-2021 (30 years), 1987-2021 (35 years), 1982-2021 (40 years), 1972-2021 (40 years), 1960-2021 (62 years), 1951-2017 (67 years).   For each time span, the procedure was as follows:   (1) For every IS two stations were identified: the nearest SY station and the nearest CL station (SY1 and CL1, Euclidean distance). Some ISs have the same SY1 or CL1 station. 9 synoptic and 11 climatological stations were identified at this step. The average distance from the IS station to the SY1 / CL1 station was 26.2 / 30.5 km; (2) Daily air temperature and daily precipitation data were downloaded for all SY1 and CL1 stations selected in (1); (3) Based on the data from the SY1 and CL1 stations, the series of climate characteristics of air temperature and precipitation were derived: the mean annual temp., the mean winter (Nov-Apr) temp., the minimum winter temp., the annual number of days with negative temp., the annual precipitation total, the annual maximum daily precip. total; (4) For each series of climate characteristics derived in (3), the one-sided hypothesis about trend was formulated and verified using the MK test.   The procedure (1-4) was repeated for every time span.  In results, the following trends were detected: the increasing in the mean annual temp. (all stations), increasing in the mean winter temp. (almost all stations), decreasing in the number of days with negative temp (50% of the stations), and increasing in the min winter temp. (several stations). No trend was observed in precipitation series.  In the paper, we described the results only for SY1 stations as they were similar at the SY1 and CL1 stations.   (b) water temp. – such data are not common in the IMGW-PIB databases. We identified only several stations (among the all studied stations) with water temperature data. Only three of them have full data records in 1992:2021. The mean annual winter temperature and the mean winter temperature were analysed. The increasing trend was detected in both variables (one station), in the mean annual temp. (two stations), and in the mean winter temp. (one stations).   The results were placed in the Results section.  For the other time-spans, the water temperature records were incomplete. |
| The occurrence and development of ice phenomena on rivers are also exerted by local environmental factors, such as the structure of the river bed, the river gradient, and underground water supplies of the rivers, and anthropogenic factors, for example, the channelling and regulation of rivers and the erection of dams and hydropower plants. The analyses and discussion of the influence of these factors to ice phenomena are very meaningful, but the paper only considered the potential impact of dam reservoirs by simply describing the distances between the crosssections downstream of the dam reservoirs. The conclusion in lines 367 to 373 ”The effect of the dams' operation on the duration of ice cover is rather negligible, so the decreasing trends in the number of days with ice, estimated in this paper, can be assigned, to a great extent, to climate change ” are insufficient and not convincing. | Indeed, the ice phenomena formation and existence depend on many factors, that we did not mention in the paper. However, such a detailed analysis of the morphology of rivers and its influence on ice phenomena would require extensive in-field investigation, that would be impossible to be done for such a large number of cross-sections as we analysed in the research (we in fact analysed over 1000 cross sections across the whole Poland, but due to the reasons described in the text we concentrated on the southern part of the country only). Our aim was to provide the information on ice phenomena at least in regional scale, but such a detailed analysis would be justified for a small single river or part of larger one.  We decided to refer to the artificial reservoirs as a factor that influences the ice formation after the literature research (where this was identified as a key issue) and due to the abundance of dams in the southern Polish rivers. Additionally, the ice jams often constitute serious problems at the artificial reservoirs. We are aware that each of the dams constitutes separate case (different altitude, engineering construction, role, area and depth of the lake, land cover, etc), and again the analysis of these factors lie beyond scope of the article. Nevertheless, we referred to the climatic conditions which confirmed that the air temperature is the most influential aspect that correlates with the annual number of days with ice phenomena. |
| Figures and tables are of low quality. | Indeed, the image quality in the PDF produced by the PLOS ONE’s submission system is poor, but we have no influence on it. Therefore, the system provides the possibility to download the figures in their full resolution (we previously uploaded figures in 600 dpi tiff format) – see the link in the top corner of the page by each figure. Nevertheless, the figures were corrected, redesigned and uploaded in 300 dpi resolution. |

Reviewer #5

| Remark | Answer |
| --- | --- |
| The Introduction is quite long. I recommend dividing this chapter into subchapters, such as a global overview, local perspective, or dividing it into two thematic sections: the effect of climate change on ice phenomena and human activities on ice phenomena. | The "Introduction" section has been significantly revised in line with reviewers' comments. We shortened the introduction by removing unnecessary fragments poorly related to the scope of research and arranged the issues so that we first presented a global overview and then a local perspective. We have corrected and clarified the results of the research described in the literature review, we have added appropriate numerical values confirming the presented conclusions from the discussed articles. We paid particular attention to a precise review of the literature regarding Poland. We also added information about new methodological approaches used lately for detecting trends in hydro-meteorological variables. Finally, we have changed the goal of the paper to be consistent with the reviewers' comments and with the expanded scope of research in the new version of the article. |
| In lines 285-287, please clarify the significance level of 'p.' | The paragraph was reformulated to make clear for the reader that we obtained such p-values when we tested the hypotheses about the Spearman correlation coefficients i.e. that it is equal to zero against greater (less) than zero. |
| Regarding the title of Figure 4, it mentions 'frequency,' but in Figure 4, the data represents the frequency, not the number of days. Please explain these differences. | The y-axis title was changed to: number of gauging cross-sections |
| In line 344, it mentions "mid-22ntr century..." – is this accurate? | Obviously, the Reviewer is right – 21^st^ century – corrected. |
| Lines 376-380: I suggest adding references to support the information presented. | We analysed also the air and water temperature and within the context of ice phenomena occurrence in the rivers that supports our opinion. Besides, in the revised version of the article we concentrated on southern part of Poland only, so this sentence has been slightly re-phrased to draw the readers’ attention to the selected, and yet specific, part of the country. |
| Lines 435-436: The conclusion seems too restrictive. The authors did not analyze cross-sections just below dams; only a few kilometers below the dam's cross-sections were analyzed. The influence of human activities, such as artificial water reservoirs, can be detected for cross-sections that are affected by water management in reservoirs, as seen in Dunajec - Sromowce Wyzne or San-Lesko. These cross-sections were not analyzed. Moreover, an interesting question is how other human activities influence ice phenomena, such as regulations of river cross-sections. | Indeed, we referred to these cross-sections, because they comply with the criteria of selection we had set in the paper.  We are aware that the ice phenomena formation and existence depend on many factors, that we did not mention in the paper. However, such a detailed analysis of the morphology of rivers and its influence on ice phenomena would require extensive in-field investigation, that would be impossible to be done for such a large number of cross-sections as we analysed in the research (we in fact analysed over 1000 cross sections across the whole Poland, but due to the reasons described in the text we concentrated on the southern part of the country only). Our aim was to provide the information on ice phenomena at least in regional scale, but such a detailed analysis would be justified for a small single river or part of larger one.  We decided to refer to the artificial reservoirs as a factor that influences the ice formation after the literature research (where this was identified as a key issue) and due to the abundance of dams in the southern Polish rivers. Additionally, the ice jams often constitute serious problems at the artificial reservoirs. |

Reviewer #6

| Remark | Answer |
| --- | --- |
| The abstract contains generic statements in lines 31-37, which should be replaced with actual findings such as trend rates to provide more specific information. | The title of the article and the abstract have been changed significantly, both in response to the reviewers' comments as well as in order to adapt to the extended scope of studies in the new version of the article. An analysis of meteorological factors, such as annual and winter (Nov-Apr) air temperature, precipitation, and water temperature, was carried out. Moreover, the relationships between the occurrence of ice phenomena and the above-mentioned factors were examined. We believe that the revised title and abstract better describe the current study. Several numerical results have been added to the abstract. Generic statements and those weakly related to actual research have been removed. |
| Throughout the literature, qualitative statements are made without accompanying values, as seen in lines 52, 54, 60, 63, 79, 91, 97-120, etc. These statements should be enhanced by including relevant values to convey the intensity of changes | The "Introduction" section has been significantly revised in line with reviewers' comments. We shortened the introduction by removing unnecessary fragments poorly related to the scope of research and arranged the issues so that we first presented a global overview and then a local perspective. We have corrected and clarified the results of the research described in the literature review, we have added appropriate numerical values confirming the presented conclusions from the discussed articles. We paid particular attention to a precise review of the literature regarding Poland. We also added information about new methodological approaches used lately for detecting trends in hydro-meteorological variables. Finally, we have changed the goal of the paper to be consistent with the reviewers' comments and with the expanded scope of research in the new version of the article. |
| Line 86-The research on ice ……… interesting facts. It does not contribute substantively to the paragraph and can be omitted | The sentence has been deleted and the relevant information has been included to other parts of the text. |
| Line 126 requires a clearer articulation of the objective. | See the comment above, please. The goal of the paper has been changed. |
| Lines 127-130 should be integrated into the data and methods section. | The whole paragraph was rephrased to better identify new goal of th research. |
| It is advisable to incorporate a brief section on the study area, including information on topography and climate. | We added a short description of the study area. The information on the air temperatures of the area were shortly presented in table A1, too. |
| Figure 1 could benefit from the inclusion of a climate graph depicting average monthly temperature (minimum, maximum, mean) and precipitation to provide readers with a visual representation of the study area's climate conditions. | We agree that such information shall be valuable for readers. Because of lack of place in Fig. 1, we added two columns to the table A1 where the mean annual temperature and the mean winter temperature for all stations were provided. |
| Latitude and longitude must be included in all the maps. | Thank you for suggesting this. The geographical coordinates were included in the maps. |
| The conclusion should be rewritten to provide clarity with specific statements rather than generic ones. Additionally, it should have the limitations and implications of the study. | The conclusion has been significantly changed to better address the amended manuscript. |

Reviewer #7

| Remark | Answer |
| --- | --- |
| Probably, the abstract could be shorter. | The abstract has been shortened. The title of the article and the abstract have been changed significantly, both in response to the reviewers' comments as well as in order to adapt to the extended scope of studies in the new version of the article. We believe that the revised title and abstract better describe the current study. |
| In the chapter 2, Please, add there the paragraph 2.1. The site description (or research area etc.). Here you should include the basic geographic and climate characterisitcs (Koppen classification, precipitation, temperature characteristics etc.), and basic morphological parameters of the studied catchments. | We thank the Reviewer for this remark. We shortly described the region accordingly on section 2, but it was not necessary to build new section devoted for the description of the region only. |
| In 2.2. Methods of the selection of gauging stations included in the analysis. There should be considered the global climate change and also disturbances in the catchments related with the civilization development (probably, those catchments should be excluded from the analysis). | Please note that our article considers the regional analysis of the ice phenomena and is based on the Polish national monitoring service datasets. We are aware that the ice phenomena formation and existence depend on many factors, that we did not mention in the paper. However, such a detailed analysis of the morphology of rivers, lad cover, disturbances and its influence on ice phenomena would require extensive in-field investigation, that would be impossible to be done for such a large number of cross-sections as we analysed in the research (we in fact analysed over 1000 cross sections across the whole Poland, but due to the reasons described in the text we concentrated on the southern part of the country only). Our aim was to provide the information on ice phenomena at least in regional scale, but a more detailed analysis would be justified for a small single river or part of larger one.  We also referred our results to local climatic conditions. The climate characteristics such as mean annual temp. and mean winter temp. from the nearest synoptic stations (years 1992:2021) were added in Table A1.  Moreover, the trend analysis was carried out for (a) air temperature and precipitation, and (b) water temperature.   (c) air temperature and precipitation data are available from two kinds of stations through the IMGW-PIB repository: the synoptic (SY) and the climatological (CL) stations. The network of the SY and CL stations is relatively dense in the study area. As stated in the Section 2.1 of the paper, there were 40 ‘ice stations’ (IS – station where ice was observed) with data from the period 1992-2021, 39 ISs with data from 1987-2021, etc. Therefore, we used the SY and CL data for every time span, namely 1992-2021 (30 years), 1987-2021 (35 years), 1982-2021 (40 years), 1972-2021 (40 years), 1960-2021 (62 years), 1951-2017 (67 years).   For each time span, the procedure was as follows:   (5) For every IS two stations were identified: the nearest SY station and the nearest CL station (SY1 and CL1, Euclidean distance). Some ISs have the same SY1 or CL1 station. 9 synoptic and 11 climatological stations were identified at this step. The average distance from the IS station to the SY1 / CL1 station was 26.2 / 30.5 km; (6) Daily air temperature and daily precipitation data were downloaded for all SY1 and CL1 stations selected in (1); (7) Based on the data from the SY1 and CL1 stations, the series of climate characteristics of air temperature and precipitation were derived: the mean annual temp., the mean winter (Nov-Apr) temp., the minimum winter temp., the annual number of days with negative temp., the annual precipitation total, the annual maximum daily precip. total; (8) For each series of climate characteristics derived in (3), the one-sided hypothesis about trend was formulated and verified using the MK test.   The procedure (1-4) was repeated for every time span.  In results, the following trends were detected: the increasing in the mean annual temp. (all stations), increasing in the mean winter temp. (almost all stations), decreasing in the number of days with negative temp (50% of the stations), and increasing in the min winter temp. (several stations). No trend was observed in precipitation series.  In the paper, we described the results only for SY1 stations as they were similar at the SY1 and CL1 stations.   (d) water temp. – such data are not common in the IMGW-PIB databases. We identified only several stations (among the all studied stations) with water temperature data. Only three of them have full data records in 1992:2021. The mean annual winter temperature and the mean winter temperature were analysed. The increasing trend was detected in both variables (one station), in the mean annual temp. (two stations), and in the mean winter temp. (one stations).   The results were placed in the Results section.  For the other time-spans, the water temperature records were incomplete. |
| and 2.3. Methods of data processing. I would recommend to include also trends in the air temperature observed in the relevant climate stations to document the regional warming. | Thank you for this suggestion. We included the trend study of the air temperature, precipitation, and water temperature. The details are below.  Air temperature and precipitation data are available from two kinds of stations through the IMGW-PIB repository: the synoptic (SY) and the climatological (CL) stations. The network of the SY and CL stations is relatively dense in the study area. As stated in the Section 2.1 of the paper, there were 40 ‘ice stations’ (IS – station where ice was observed) with data from the period 1992-2021, 39 ISs with data from 1987-2021, etc. Therefore, we used the SY and CL data for every time span, namely 1992-2021 (30 years), 1987-2021 (35 years), 1982-2021 (40 years), 1972-2021 (40 years), 1960-2021 (62 years), 1951-2017 (67 years).  For each time span, the procedure was as follows:   (1) For every IS two stations were identified: the nearest SY station and the nearest CL station (SY1 and CL1, Euclidean distance). Some ISs have the same SY1 or CL1 station. 9 synoptic and 11 climatological stations were identified at this step. The average distance from the IS station to the SY1 / CL1 station was 26.2 / 30.5 km; (2) Daily air temperature and daily precipitation data were downloaded for all SY1 and CL1 stations selected in (1); (3) Based on the data from the SY1 and CL1 stations, the series of climate characteristics of air temperature and precipitation were derived: the mean annual temp., the mean winter (Nov-Apr) temp., the minimum winter temp., the annual number of days with negative temp., the annual precipitation total, the annual maximum daily precip. total; (4) For each series of climate characteristics derived in (3), the one-sided hypothesis about trend was formulated and verified using the MK test.   The procedure (1-4) was repeated for every time span.  In results, the following trends were detected: the increasing in the mean annual temp. (all stations), increasing in the mean winter temp. (almost all stations), decreasing in the number of days with negative temp (50% of the stations), and increasing in the min winter temp. (several stations). No trend was observed in precipitation series.  In the paper, we placed the results only for SY1 stations as they were similar at the SY1 and CL1 stations.  We also analysed water temperature. However, such data are not common in the IMGW-PIB databases. We identified only several stations (among the all studied stations) with water temperature data. Only three of them have full data records in 1992:2021. The mean annual winter temperature and the mean winter temperature were analysed. The increasing trend was detected in both variables (one station), in the mean annual temp. (two stations), and in the mean winter temp. (one stations). |
| In the Discussion (chapter 4), authors should discuss also the rate of global warming (trends in air temperature), and to include future scenarios related to the future ice phenomena on the rivers considered. | In the revised manuscript we added a new reference (Kundzewicz and Matczak, 2012) who deal with regional differences of climate change impacts, to highlight that our results are in line with other studies on regional aspects of climatic change in Poland. |
| In Conclusions (chapter 5) , please add there some numbers (results) corresponding with the outcomes of your paper. | In the revised paper we included the key values obtained in our study, which made the conclusions paragraph more informative. |

Reviewer #8

| Who | Nr | Remark | Answer |
| --- | --- | --- | --- |
| IKM | 1 | **the summary requires improvement, it should contain a short description of the purpose of the research, the methods used, the research area and the results obtained**  the introduction of the work requires improvement, it is necessary to refer to the gaps in the literature and indicate what is new in the work and what research gap has been filled. | The title of the article and the abstract have been changed significantly, both in response to the reviewers' comments as well as in order to adapt to the extended scope of studies in the new version of the article. An analysis of meteorological factors, such as annual and winter (Nov-Apr) air temperature, precipitation, and water temperature, was carried out. Moreover, the relationships between the occurrence of ice phenomena and the above-mentioned factors were examined. We believe that the revised title and abstract better describe the current study. Several numerical results have been added to the abstract. Generic statements and those weakly related to actual research have been removed. |
| Kk+mg | 2 | There is a shortage at work clearly defined main purpose of the work and specific goals. Please indicate clearly whether days with ice phenomena were analyzed (including coastal ice, frigid ice, ice cover, floes) or only days with ice cover.  Moreover, it should be clarified what the term "5-10, 15-20 years or more" means recently? (line125-126).  The presented literature requires supplementation. There are no studies by Marek Grześ, who perfectly described the conditions for the formation of ice phenomena on the lower Vistula, and by other authors. | We analysed all the days, with any ice phenomena occurred (grease, floe, shore ice, ice cover, ice jam, etc). Indeed, it was not mentioned and now was clarified it in the introduction and in the second chapter.  We corrected spelling in this sentence.  Indeed, the papers by prof. Marek Grześ considered the interesting aspects of ice jams in the Lower Vistula River and the formation of ice phenomena (Grześ, 1989). We, however, decided not to cite them, because the journals in which these works were published might be difficult to access by foreign audience. |
| kk | 3 | the description of the research methodology requires improvement, and especially subsection 2.1 needs to be shortened. You should briefly describe on what basis the data was selected, without going into details. In its current form, some of the information in this subsection is redundant, e.g. the description of the IMWM database. | We think that that the selection of the datasets is crucial for further analysis. Since it may seem complex at the first sight, we decided to describe it thoroughly. Also, we treat the paper as the introductory research on the ice phenomena in Poland based on the information ‘hidden’ in the IMWM’s repositories (counting on correction of the mistakes in the northern cross sections in the future), so a short presentation on what these repository offers is necessary. |
| Kk+KBG  +mr+ar | 4 | please explain why the authors presented data for 5 hydrological stations with the longest observation sequences. These data are difficult to compare due to the different start and end dates of observations –  **The authors analyzed only one parameter - the duration of ice phenomena, they did not analyze the start and end dates of ice phenomena and individual types of ice phenomena, which I consider a significant simplification.** | We decided to show the results for these data because they provide the insight into long-term trends in the ice phenomena occurrence. Note that the periods covered by these datasets intersect in large parts.  We fully agree with the reviewer’s point of view that introducing the start and end dates could yield interesting insights to the ice studies. Nevertheless, this is a much broader issue that could make the paper unconcise. We plan to make such a study in the future. |
| IKM | 5 | **the work contains too few references to changes in meteorological conditions in individual regions of Poland. Somorowska's work covers only the small catchment area of the Liwiec River. There is a lack of newer literature that will allow for comparison of trends in air temperature changes, especially including data for the reference period 1991-2020 and covering much larger areas.** | The "Introduction" section has been significantly revised in line with reviewers' comments. We shortened the introduction by removing unnecessary fragments poorly related to the scope of research and arranged the issues so that we first presented a global overview and then a local perspective. We have corrected and clarified the results of the research described in the literature review, we have added appropriate numerical values confirming the presented conclusions from the discussed articles. We paid particular attention to a precise review of the literature regarding Poland. Some references refer to changes in meteorological conditions. We also added information about new methodological approaches used lately for detecting trends in hydro-meteorological variables. Finally, we have changed the goal of the paper to be consistent with the reviewers' comments and with the expanded scope of research in the new version of the article.  We included the trend study of the air temperature, precipitation, and water temperature. The details are below.  Air temperature and precipitation data are available from two kinds of stations through the IMGW-PIB repository: the synoptic (SY) and the climatological (CL) stations. The network of the SY and CL stations is relatively dense in the study area. As stated in the Section 2.1 of the paper, there were 40 ‘ice stations’ (IS – station where ice was observed) with data from the period 1992-2021, 39 ISs with data from 1987-2021, etc. Therefore, we used the SY and CL data for every time span, namely 1992-2021 (30 years), 1987-2021 (35 years), 1982-2021 (40 years), 1972-2021 (40 years), 1960-2021 (62 years), 1951-2017 (67 years).  For each time span, the procedure was as follows:   (1) For every IS two stations were identified: the nearest SY station and the nearest CL station (SY1 and CL1, Euclidean distance). Some ISs have the same SY1 or CL1 station. 9 synoptic and 11 climatological stations were identified at this step. The average distance from the IS station to the SY1 / CL1 station was 26.2 / 30.5 km; (2) Daily air temperature and daily precipitation data were downloaded for all SY1 and CL1 stations selected in (1); (3) Based on the data from the SY1 and CL1 stations, the series of climate characteristics of air temperature and precipitation were derived: the mean annual temp., the mean winter (Nov-Apr) temp., the minimum winter temp., the annual number of days with negative temp., the annual precipitation total, the annual maximum daily precip. total; (4) For each series of climate characteristics derived in (3), the one-sided hypothesis about trend was formulated and verified using the MK test.   The procedure (1-4) was repeated for every time span.  In results, the following trends were detected: the increasing in the mean annual temp. (all stations), increasing in the mean winter temp. (almost all stations), decreasing in the number of days with negative temp (50% of the stations), and increasing in the min winter temp. (several stations). No trend was observed in precipitation series.  In the paper, we placed the results only for SY1 stations as they were similar at the SY1 and CL1 stations.  We also analysed water temperature. However, such data are not common in the IMGW-PIB databases. We identified only several stations (among the all studied stations) with water temperature data. Only three of them have full data records in 1992:2021. The mean annual winter temperature and the mean winter temperature were analysed. The increasing trend was detected in both variables (one station), in the mean annual temp. (two stations), and in the mean winter temp. (one stations). |
| mg | 6 | discussions should be improved, referred to the results obtained and compared with other authors. The occurrence of ice phenomena is influenced not only by air temperature but also by the shape of the cross-section and the flow rate. Anthropogenic factors include the location of the water gauge station in relation to the dam and the location of discharges of polluted, saline or heated water. Apart from air temperature, the authors do not mention any other factors that influence the formation of ice phenomena | In the revised version of our paper we improved the clarity of the message and referred our results to a range of published materials. We also discussed the issues of human influences. However, the main goal of our paper was to document the trends in ice phenomena, putting the reasons of the documented changes aside and speculative. All of the issues responsible for ice phenomena formation were listed in the discussion and addressed to an extent that our study could do it. We hope that the revised paper better addresses the scope of the paper. |
| Kk+mg | 7 | conclusions should refer only to your own findings reported in the article. e.g. the authors did not prove in their study that "the analysis of data on ice phenomena in southern Poland showed a large impact of climate change on the ice cover of the temperate climate zone" | Thank you for this suggestion – we corrected the conclusions |
| Kk+mg+native? | 8 | English requires verification by a native speaker. Some wording requires correction, e.g. the entry on page 3, line 62-65 |  |
| kk | 9 | the figures attached at the end of the article have not been signed | These figures was automatically placed at the end of the article by the PLOS ONE’s submission system. Note that at the upper right corner there is a link to full version of the figure with its number allowing for the identification of the figure. The uploading system does not provide possibility to add captions in this place. |
| KBG | 10 | **the figures are of poor quality,** most of the rivers are not marked on the map, which makes identification impossible for readers from outside Poland | Indeed, the image quality in the PDF produced by the PLOS ONE’s submission system is poor, but we have no influence on it. Therefore, the system provides the possibility to download the figures in their full resolution (we previously uploaded figures in 600 dpi tiff format) – see the link in the top corner of the page by each figure. Nevertheless, the figures were corrected, redesigned and uploaded in 300 dpi resolution. For the sake of map clarity, only the main rivers of Poland (the Vistula and the Oder) and their major tributaries have been marked on the maps. Because many gauge stations are located at rivers that are tributaries of tributaries of these rivers - it is not possible to mark these rivers on this scale. |

Reviewer #9

| Who | Nr | Remark | Answer |
| --- | --- | --- | --- |
| mg | 1 | General feedback: The article employed the simplest method, compiling data from relatively long series to analyze changes in the ice trend. However, there is a lack of supporting data for the causes of this phenomenon, particularly in the discussion section, where there is no discussion based on the existing results. Instead, there is an excessive focus on climate change and ecological impacts not covered in this article. | We thank the Reviewer for this suggestion. In order to refer to the supporting data we included the trend study of the air temperature, precipitation, and water temperature. |
| Kk+mr | 2 | line 31“Using straightforward, but commonly accepted procedures…”Highlight the advantages of choosing this method, rather than selecting it merely because it is commonly used. | We chose this method because it is commonly used and understandable, so our results are more likely to be compared to the results by other authors. |
| IKM | 3 | **lines 36-38 are speculative passage, and including them in the abstract may give the impression of padding. I recommended to either remove them or condense them into a single sentence conveying the research significance.** | The title of the article and the abstract have been changed significantly, both in response to the reviewers' comments as well as in order to adapt to the extended scope of studies in the new version of the article. An analysis of meteorological factors, such as annual and winter (Nov-Apr) air temperature, precipitation, and water temperature, was carried out. Moreover, the relationships between the occurrence of ice phenomena and the above-mentioned factors were examined. We believe that the revised title and abstract better describe the current study. Several numerical results have been added to the abstract. Generic statements and those weakly related to actual research have been removed. |
| KBG+AR | 4 | **lines 358-360,“we revealed that it was the last 30…...” and lines 377-378,“we revealed that it …..” This specific conclusion lacks solid temperature data support, it is suggested to supplement with relevant climate data or water temperature data**. | Thank you for this suggestion. We included the trend study of the air temperature, precipitation, and water temperature. The details are below.  Air temperature and precipitation data are available from two kinds of stations through the IMGW-PIB repository: the synoptic (SY) and the climatological (CL) stations. The network of the SY and CL stations is relatively dense in the study area. As stated in the Section 2.1 of the paper, there were 40 ‘ice stations’ (IS – station where ice was observed) with data from the period 1992-2021, 39 ISs with data from 1987-2021, etc. Therefore, we used the SY and CL data for every time span, namely 1992-2021 (30 years), 1987-2021 (35 years), 1982-2021 (40 years), 1972-2021 (40 years), 1960-2021 (62 years), 1951-2017 (67 years).  For each time span, the procedure was as follows:   (1) For every IS two stations were identified: the nearest SY station and the nearest CL station (SY1 and CL1, Euclidean distance). Some ISs have the same SY1 or CL1 station. 9 synoptic and 11 climatological stations were identified at this step. The average distance from the IS station to the SY1 / CL1 station was 26.2 / 30.5 km; (2) Daily air temperature and daily precipitation data were downloaded for all SY1 and CL1 stations selected in (1); (3) Based on the data from the SY1 and CL1 stations, the series of climate characteristics of air temperature and precipitation were derived: the mean annual temp., the mean winter (Nov-Apr) temp., the minimum winter temp., the annual number of days with negative temp., the annual precipitation total, the annual maximum daily precip. total; (4) For each series of climate characteristics derived in (3), the one-sided hypothesis about trend was formulated and verified using the MK test.   The procedure (1-4) was repeated for every time span.  In results, the following trends were detected: the increasing in the mean annual temp. (all stations), increasing in the mean winter temp. (almost all stations), decreasing in the number of days with negative temp (50% of the stations), and increasing in the min winter temp. (several stations). No trend was observed in precipitation series.  In the paper, we placed the results only for SY1 stations as they were similar at the SY1 and CL1 stations.  We also analysed water temperature. However, such data are not common in the IMGW-PIB databases. We identified only several stations (among the all studied stations) with water temperature data. Only three of them have full data records in 1992:2021. The mean annual winter temperature and the mean winter temperature were analysed. The increasing trend was detected in both variables (one station), in the mean annual temp. (two stations), and in the mean winter temp. (one stations). |
| kk | 5 | lines 367-371,“we revealed that it was the last 30 years that experienced most……”I suggest you present the results divided into situations with and without a reservoir, instead of just listing them simply |  |
| mg | 6 | lines 392-429. The entire article does not contain any data related to biology, but the author wastes a lot of space discussing ecological impacts. I suggest Reduce the description in this regard, and focus on comparing and analyzing the reasons and patterns causing the differences in your results on a global scale. | We rephrased parts of the discussion mentioned by the reviewer. We added information about the possible societal and economic consequences (to our best knowledge – mainly positive). Mentioning the ecological issues was considered important as mainly the environmental factors can be impaired by the changing ice phenomena. We suggest to keep the revised discussion as is. |

Reviewer #10

| Who | Nr | Remark | Answer |
| --- | --- | --- | --- |
| IKM | 1 | **The title does not match the content of the article. The authors selected observation posts for analysis located in southern Poland, mainly on mountain rivers. Additionally, data series were analyzed for 5 stations located on the Vistula River. The database does not represent the entire country. I suggest changing the title of the article!** | The title of the article, abstract and the text have been changed significantly, both in response to the reviewers' comments as well as in order to adapt to the extended scope of studies in the new version of the article. An analysis of meteorological factors, such as annual and winter (Nov-Apr) air temperature, precipitation, and water temperature, was carried out. Moreover, the relationships between the occurrence of ice phenomena and the above-mentioned factors were examined. We believe that the revised title and abstract better describe the current study. |
| IKM | 2 | **The authors' wording in the Abstract is at least surprising, there are many publications describing the results of temporal and spatial changes in the occurrence of ice phenomena have been conducted in temperate regions. When preparing the article, the authors read many publications on this topic, as evidenced by the References list.** | Please see the comment above. |
| IKM | 3 | **In the introduction, the authors did not present the issue of methods for determining the trends in changes in ice phenomena, which is the subject of their analysis. Currently, research is also being undertaken using new methodological approaches, e.g. machine learning and artificial intelligence. There is not even a mention of the methods, and there is a lot of information about the importance of the results for the needs of water management, which the authors practically did not deal with in the article.** | The "Introduction" section has been significantly revised in line with reviewers' comments. We shortened the introduction by removing unnecessary fragments poorly related to the scope of research and arranged the issues so that we first presented a global overview and then a local perspective. We have corrected and clarified the results of the research described in the literature review, we have added appropriate numerical values confirming the presented conclusions from the discussed articles. We paid particular attention to a precise review of the literature regarding Poland. Some references refer to changes in meteorological conditions. We also added information about new methodological approaches used lately for detecting trends in hydro-meteorological variables |
| IKM | 4 | **The authors defined the goal for the article: "The main objective was to answer the question if the river ice phenomena have been decreasing over the last decades, as it is commonly considered". However, it seems that this hypothesis has been confirmed for Polish and European rivers quite a long time ago**. | We have changed the goal of the paper to be consistent with the reviewers' comments and with the expanded scope of research in the new version of the article. |
| IKM | 5 | **The methods used in the work are indeed quite widely accepted, so it can be concluded that the article is not a novelty in the field of research on ice phenomena.** | As it is written in the paragraph about the goal of the paper, the main objective of the paper was to study changes in the occurrence of ice phenomena in upland and mountain regions of Poland due to meteorological conditions, as there is a lack of research on ice phenomena in such regions. Hence the emphasis was put on environmental aspect of research and commonly accepted statistical method, i.e. the Mann-Kendall test with Bonferroni correction, was used to study the temporal changes in the annual number of days with ice. |
| kk | 6 | For what purpose was one station with data for the period 1951-2021 analyzed? Is this a representative station for Poland? | This was the only station with such a long data series available for the analysis. We decided to highlight this station for comparative purposes. |
| kk | 7 | According to the reviewer, the information in lines 144-148 is unnecessary; they are not relevant to the analysis. | We treat this paper as the introductory research on the ice phenomena in Poland based on the information ‘hidden’ in the IMWM’s repositories (counting on correction of the mistakes in the northern cross sections in the future), so a short presentation on what these repository offers is necessary. |
| kk | 8 | The authors adopted the annual number of days with ice as the key variable. How should "with ice" be interpreted - is it any ice phenomenon or a permanent ice cover? This is important because the number of days with ice cover is much smaller than the number of days with all ice phenomena. | Indeed, such information is missing and yet crucial. Thank you. We analysed all the days, if any ice phenomena occurred (grease, floe, shore ice, ice cover, ice jam, etc). Indeed, it was not mentioned and now was clarified it in the introduction and in the second chapter. |
| kk | 9 | Note to the description in line 153: Many studies provide the dates of the beginning of ice phenomena on Polish rivers. On what basis did the authors draw such a conclusion? How were these dates verified as incorrect? Was method used to assess the homogeneity of the IMGW-PIB observational series? In this paper, the authors only analyze the number of days - descriptive statistics. The phenomenon exists or does not exist, and the dates of occurrence of ice phenomena have not been studied. | First of all, after noticing the ‘ice in summer’ datasets we contacted the person IMGW-PIB responsible for these datasets to verify our suspicions. Besides, there is strong correlation between the air temperature and occurrence of ice phenomena in rivers. |
| Kk?  IKM | 10 | The occurrence of ice phenomena on mountain rivers differs from the rest of the country. Climatic conditions - especially air temperature and water temperature, affecting the ice regime of rivers - are different than in the lowland regions and coastal regions of Poland. Therefore that the title of the work is not adequate to the content of the work. It should be taken into account that the lowlands in Poland cover 91.3% of the area, the highlands 5.6%, and the mountains 3.3%, of which 0.2% is high mountains. The authors write: The selected gauging stations are located mostly in the Carpathian Mountains, in the southern part of Poland, leaving vast areas beyond our investigation". | Thank you very much for this remark. The title has been changed and so was the aim of the research to address more adequately the research based practically only on the datasets for southern Polish rivers. |
| kk | 11 | In the _pdf file, page 11 is not filled with content. This is an editorial error, whether some information is missing? - it's hard to guess. Similarly, page 13? | Corrected. |
| IKM | 12 | **Table 2 to modify. I suggest removing the columns for 50 years. and describe Ropa/Klęczany in the text. Empty spaces are not conducive to good reception of information.** | The Table 2 has been corrected, i.e. the almost empty columns have been removed, and information about the trend for the 50-element data series has been added in the text of the manuscript. |
| kk | 13 | Note to lines 364-366: There is no basis for such formulations because the authors did not carry out any forecast of changes in the number of ice phenomena on rivers. The obtained results only determine the number of days with ice and determine trends. Trend is not equivalent to forecast! | We are aware of this fact. That is why, in the revised manuscript we stated that “if the observed trend continued, we might expect ice phenomena to disappear completely from rivers in southern Poland by 2040-2070.” This is not a forecast but a solid statement provided based on the appropriate assumption. |
| kk | 14 | Note to lines 367 - this was not the subject of the authors' research, nor was it the purpose of the authors' research. Only in the Discussion did the authors refer to this issue. |  |
| kk | 15 | Note on line 376: Determining regional patterns requires increasing the number of observations outside the area studied by the authors! The authors only studied rivers in southern Poland. In the discussion, the authors assume a number of scenarios of continued trends in changes or disappearance of ice phenomena on rivers, referring to the causative factors, but no such analyzes were carried out in the study. The conclusions are only assumptions and are based on the results of previous research conducted in Poland. | Thank you for this remark. In the rephrased conclusions we managed to state clearly the values we obtained in our study. We believe the improved discussion and conclusions are now in line with the materials and methods applied. |
| kk | 16 | The discussion does not refer to the results obtained; It's not exactly on the right top. | We rephrased the discussion. We added the elements of socio-economic consequences of disappearing ice phenomena as well as discussed the negative consequences, whose origin is primarily ecologic. |
