# Supplementary material for: Analysis of changes in the occurrence of ice phenomena in upland and mountain rivers of Poland
Source: PLoS One. 2024 Jul 26;19(7):e0307842. doi: 10.1371/journal.pone.0307842 (PMC11280165; doi:10.1371/journal.pone.0307842)
Supplement: Attachment — Submitted filename: comment 9.docx [file pone.0307842.s006.docx]

The author highly respects the opinions of the reviewers, and I suggest receiving after after minor revision. The author has greatly revised and comprehensively improved the article, but there are still the following areas worthy of improvement:

1. The abbreviated NID in line 28 should be placed in front of the word “phenomena”

2. Lines 65 to 82 are the summary of the research area, which is very good, but there is no summary of the common points and differences of the research, just a simple enumeration of the literatures. The purpose of writing the research background is to extend the research necessity of the author's article, so I hope the author can summarize the article when listing the articles.

3.Line 331 number of days with ice can be abbreviated to NID, and similar problems in other parts of the article are corrected.

4. The full text has an grammatical tense disorder. I suggest to check and revise the full text.
