# Supplementary material for: Analysis of changes in the occurrence of ice phenomena in upland and mountain rivers of Poland
Source: PLoS One. 2024 Jul 26;19(7):e0307842. doi: 10.1371/journal.pone.0307842 (PMC11280165; doi:10.1371/journal.pone.0307842)
Supplement: Attachment — Submitted filename: Answer to Reviewers after second reviews June 2024.docx [file pone.0307842.s007.docx]

**Answers to the Reviewers’ remarks**

Dear Editors, dear Reviewers

We would like to thank very much Prof. Seema Rani, Prof. Ting Li and other anonymous Reviewers for the remarks and your valuable time you devoted to review our paper. We believe, that thanks to your remarks the new version will meet the high standards of the PLOS ONE and will be ready for publication. In the following document we concentrate only on the critical remarks answering to them and changing manuscript accordingly. At the same time we expres our appreciation for good words about our work.

We count that the new version of the manuscript will satisfy both the Reviewers, Editors and finally the readers.

With best regards

Yours faithfully

Authors

Reviewer #8: The article has been largely improved. Not all comments were taken into account. However, there are few references to Polish literature in the article, which may mean that the authors did not precisely recognize the essence of the problem. The authors explain that they did not quote M. Grześ's works because it makes it difficult for foreigners to read. This is in contradiction with the cited works by Paczowska (1938) and Bączyk and Suchożebrski (2012), which are in Polish. Moreover, the authors quote the article in Italian (see Bonferroni 1936).

*Answer: Thank you very much for drawing our attention to the Prof Grześ’ publications. We considered the monumental works by Prof. Grześ in new version of the article.*

Reviewer #9: The author highly respects the opinions of the reviewers, and I suggest receiving after after minor revision. The author has greatly revised and comprehensively improved the article, but there are still the following areas worthy of improvement:

1. The abbreviated NID in line 28 should be placed in front of the word “phenomena”

*Answer: Thank you very much for this suggestion; we corrected the text accordingly.*

2. Lines 65 to 82 are the summary of the research area, which is very good, but there is no summary of the common points and differences of the research, just a simple enumeration of the literatures. The purpose of writing the research background is to extend the research necessity of the author's article, so I hope the author can summarize the article when listing the articles.

*Answer: We corrected this fragment according the Reviewer’s suggestions.*

3. Line 331 number of days with ice can be abbreviated to NID, and similar problems in other parts of the article are corrected.

*Answer: We corrected the manuscript accordingly.*

4. The full text has an grammatical tense disorder. I suggest to check and revise the full text.

*Answer: We revised the article accordingly. We tried to keep the narration in past tense, unless other tense was necessary to highlight the discussed issue.*
